# Single Sustained Inflation followed by Ventilation Leads to Rapid Cardiorespiratory Recovery but Causes Cerebral Vascular Leakage in Asphyxiated Near-Term Lambs

**DOI:** 10.1371/journal.pone.0146574

**Published:** 2016-01-14

**Authors:** Kristina S. Sobotka, Stuart B. Hooper, Kelly J. Crossley, Tracey Ong, Georg M. Schmölzer, Samantha K. Barton, Annie R. A. McDougall, Suzie L. Miller, Mary Tolcos, Claus Klingenberg, Graeme R. Polglase

**Affiliations:** 1 The Ritchie Centre, Monash University, Melbourne, Australia; 2 Institute of Neuroscience and Physiology, The Sahlgrenska Academy, University of Gothenburg, Gothenburg, Sweden; 3 Department of Obstetrics and Gynaecology, Monash University, Melbourne, Australia; 4 Department of Pediatrics, Medical University, Graz, Austria; 5 Centre for the Studies of Asphyxia and Resuscitation, Neonatal Research Unit, Royal Alexandra Hospital, Edmonton, Canada; 6 Department of Pediatrics, University of Alberta, Edmonton, Canada; 7 Department of Paediatrics, University Hospital of North Norway, Tromsø, Norway; 8 Paediatric Research Group, Faculty of Health Sciences, University of Tromsø, Tromsø, Norway; Institute of Neurology (Edinger-Institute), GERMANY

## Abstract

**Background:**

A sustained inflation (SI) rapidly restores cardiac function in asphyxic, bradycardic newborns but its effects on cerebral haemodynamics and brain injury are unknown. We determined the effect of different SI strategies on carotid blood flow (CaBF) and cerebral vascular integrity in asphyxiated near-term lambs.

**Methods:**

Lambs were instrumented and delivered at 139 ± 2 d gestation and asphyxia was induced by delaying ventilation onset. Lambs were randomised to receive 5 consecutive 3 s SI (multiple SI; n = 6), a single 30 s SI (single SI; n = 6) or conventional ventilation (no SI; n = 6). Ventilation continued for 30 min in all lambs while CaBF and respiratory function parameters were recorded. Brains were assessed for gross histopathology and vascular leakage.

**Results:**

CaBF increased more rapidly and to a greater extent during a single SI (p = 0.01), which then decreased below both other groups by 10 min, due to a higher cerebral oxygen delivery (p = 0.01). Blood brain barrier disruption was increased in single SI lambs as indicated by increased numbers of blood vessel profiles with plasma protein extravasation (p = 0.001) in the cerebral cortex. There were no differences in CaBF or cerebral oxygen delivery between the multiple SI and no SI lambs.

**Conclusions:**

Ventilation with an initial single 30 s SI improves circulatory recovery, but is associated with greater disruption of blood brain barrier function, which may exacerbate brain injury suffered by asphyxiated newborns. This injury may occur as a direct result of the initial SI or to the higher tidal volumes delivered during subsequent ventilation.

## Introduction

Infants born severely asphyxic are usually bradycardic and apneic and require some form of assisted ventilation at birth. As these infants are born with liquid-filled lungs, the airways must be cleared of liquid to establish pulmonary gas exchange and to decrease pulmonary vascular resistance [1, 2]. Currently, there is no globally accepted strategy for the resuscitation of asphyxiated newborns.

Adverse cardiopulmonary events associated with neonatal resuscitation are closely related to cerebral pathology in premature newborns and can be triggered by mechanical ventilation and rapid lung volume expansion [3]. For instance, initiation of ventilation with high positive end expiratory pressures or tidal volumes adversely affects pulmonary blood flow and left ventricular preload, resulting in large changes in cardiac output, systemic and brain inflammation, oxidative stress and evidence of cerebral vascular leakage [4, 5]. Thus, poorly controlled respiratory care resulting in cardiovascular and cerebral haemodynamic instability may underlie the pathogenesis of some perinatal brain injury.

A sustained inflation (SI) at a constant pressure is very effective at aerating the lungs and rapidly restoring heart rate and blood pressures in asphyxiated bradycardic newborns [6]. Presumably this effect of a SI results from improved aeration of the distal air sacs, which not only facilitates the onset of pulmonary gas exchange but also promotes and an increase in pulmonary blood flow leading to greatly improved cardiac function and arterial oxygenation [7]. Indeed, an effective SI also improves heart rate and cerebral tissue oxygen saturation in very low birth weight human infants [8].

The International Liaison Committee on Resuscitation guidelines recommend initiating ventilation with either shorter or longer inspiratory times in bradycardic apneic term infants [2] whereas the European guidelines recommend inflation pressures of 2–3 s for the first five inflations [9]. These recommendations are mainly derived from studies demonstrating that initiating resuscitation with inflations of 2–5 s improved functional residual capacity in asphyxiated term infants [10, 11]. More recently, we found that an initial 30 s SI restores heart rate and arterial blood pressure much more rapidly in asphyxiated near-term lambs than either 5 consecutive 3 s SI or conventional ventilation [12]. However, the restoration in heart rate and blood pressure was so rapid that the resulting increase in cerebral blood flow may induce or enhance brain pathology. It is currently not known how different SI strategies may affect cerebral blood flow and oxygenation during resuscitation from severe asphyxia.

Our aim was to determine the effect of a single 30 s SI on carotid blood flow, and determine whether a SI increases the susceptibility to vascular leakage within the brain following severe asphyxia. Specifically, we compared carotid blood flow (CaBF) and cerebral histopathology from asphyxiated lambs given an initial 30 s SI, 5 consecutive 3 s SI, or conventional ventilation. We hypothesized that a 30 s SI will increase CaBF and the risk of cerebral hemorrhage.

## Methods

All experimental procedures were approved by the relevant Monash University Animal Ethics Committee in accordance with the National Health and Medical Research Council (Australia) Australian code of practice for the care and use of animals for scientific purposes (7^th^ Edition, 2004).

Pregnant ewes at 139 ± 2 (mean ± SD) days of gestation (term is ~147 days) were anesthetised and the fetal head and neck were exposed via caesarean section. Catheters were inserted into a fetal carotid artery and jugular vein. A 3 mm ultrasonic flow probe (Transonic Systems, Ithaca, NY, USA) was placed around the non-catheterised carotid artery, as described previously [13] as a surrogate measure for cerebral blood flow. Changes in carotid arterial blood flow (CaBF) are strongly correlated with changes in cerebral blood flow in other sheep models [14, 15]. The fetal trachea was intubated with a cuffed endotracheal tube (5 mm) and lung liquid was drained passively for 20 s. A transcutaneous oximeter (Masimo, Irvine, CA, USA) was attached around the right forelimb before the umbilical cord was clamped and cut.

Lambs were randomised into ventilation groups (see below) delivered, dried, weighed and placed under a radiant heater. Asphyxia was induced in the lamb by delaying initiation of ventilation until carotid mean arterial pressure had decreased to ~20 mmHg, at which time ventilation commenced. Lambs received 5% dextrose (i.v.) and were sedated (alfaxane i.v.: 5–15 mg/kg/hr; Jurox, East Tamaki, Auckland, New Zealand) as an ethical requirement. Ewes were humanely killed (sodium pentobarbitone: ~100 mg/kg, i.v.) after delivery of the lamb.

CaBF (Powerlab; ADInstruments, Castle Hill, NSW, Australia) and pressure (DTX Plus Transducer; Becton Dickinson, Singapore), tidal volume (V_T_) and airway pressure were electronically recorded throughout the experiment using a data acquisition system (Powerlab; ADI; Australia). The ventilation procedures for each group were as follows: i) 5 consecutive 3 s sustained inflations with 1 s expiratory time (multiple SI; n = 6); ii) a single 30 s sustained inflation (single SI; n = 6); or iii) conventional ventilation (no SI; n = 6). Lambs in Group 1 (multiple SI group) received a peak inspiratory pressure (PIP) of 35 cmH_2_O, a positive end expiratory pressure (PEEP) of 5 cmH_2_O and a fraction of inspired oxygen (FiO_2_) of 0.21 delivered using a mechanical ventilator (Babylog 8000+ ventilator; Drager, Lubeck, Germany). This protocol is consistent with the European neonatal resuscitation guidelines that recommend the maintenance of inflation pressure for 2–3 s for the first 5 inflations [9]. Group 2 lambs (single SI group) received a single 30 s SI delivered by a T-piece device (Neopuff; Fisher & Paykel Healthcare, Panmure, Auckland, New Zealand) with a PIP of 35 cmH_2_O, PEEP of 5 cmH_2_O and a FiO_2_ of 0.21. Following the SI, these lambs were mechanically ventilated for 10 min using a PIP of 35 cmH_2_O, PEEP of 5 cmH_2_O and a FiO_2_ of 0.21 at a rate of 60 bpm (Ti = 0.5 s and Te = 0.5 s) with heated and humidified gas. After 10 min, the lambs were ventilated using a set V_T_ of 8 mL/kg with a FiO_2_ of 0.21 for 20 min. This mechanical ventilation protocol was also followed for all lambs in group 3 (ie without the initial SI) receiving conventional ventilation.

Arterial blood samples were taken regularly after the onset of ventilation for measurement of partial pressure of arterial oxygen (PaO_2_), carbon dioxide (PaCO_2_), pH and arterial oxygen saturation (SaO_2_; ABL30, Radiometer, Copenhagen, Denmark). At 30 min after ventilation onset, lambs were killed by anaesthetic overdose (sodium pentobarbitone; 130 mg/kg i.v.) and the brains collected.

### Brain collection, staining and analysis

At autopsy, the brain was removed from the skull of lambs (multiple SI: n = 6; single SI: n = 6; and no SI: n = 6) and the cerebrum halved along the midline. The left hemisphere was cut coronally into blocks 4 mm thick (8–9 blocks per animal), post-fixed in 4% paraformaldehyde in 0.1M phosphate buffer (pH 7.4) for 3 days and embedded in paraffin. Serial, 10 μm-thick sections were cut from 3 blocks: one each at the level of the striatum, the thalamus and the anterior pole of the hippocampus (equivalent to sections 760, 960 and 1120 of The Sheep Brain Atlas; www.msu.edu/~brains/brains/sheep/index.html). Thus three different levels of the forebrain were assessed.

Two coronal sections per block (n = 6 section per animal), separated by 100μm were stained using Hematoxylin and Eosin (H & E) for orientation purposes and to assess for the presence of overt injury (hemorrhages, lesions or necrosis). One section per block (n = 3 sections per animal) was immunostained with rabbit anti-sheep serum (1:1000, Sigma, Missouri, USA, Product number S4265) to identify altered blood-brain permeability. Sections were incubated in biotinylated goat anti-rabbit IgG antibody (1:200), reacted using the avidin-biotin complex elite kit (Vector Laboratories, Burlingame, CA) and counterstained with 0.1% thionin in acetate buffer (pH 4.4) as previously described [16]. Prior to analysis all slides were coded with the observer blinded to treatment to prevent experimenter bias. Blood vessel profiles with serum extravasation were counted within the entire subcortical white matter, periventricular white matter and cortical grey matter in each of the 3 sections per animal. The mean number of vessel profiles with protein extravasation was calculated for each region (subcortical white matter, periventricular white matter, cortical grey matter) per animal, and a mean of means then determined for each treatment group.

### Physiological Analytical methods

For analysis of changes in physiology, data were divided into two periods of time (0 to 10 min and 10 to 30 min after ventilation onset) as the ventilation protocol changed at 10 min after initiation of ventilation. Epochs of 5 s were averaged every 30 s for the first 10 min. Epochs were then averaged every minute until 15 min, then at 20, 25 and 30 min. Waveform parameters (mean, mean systolic and mean diastolic flow) from CaBF were analysed. Carotid arterial pulsatility index (PI), a measure of downstream resistance to blood flow [17], was calculated as:

PI = (peak systolic CaBF – minimum diastolic CaBF) / mean CaBF

The peak first derivative of the carotid arterial blood flow pulse (peak dF/dt), a measure of heart contractility, was also analysed [18].

Carotid arterial blood O_2_ content from arterial blood gases was measured to estimate cerebral O_2_ delivery [19], according to the formula:

$${\text{O}}_{2}\text{ delivery }=\text{ blood }{\text{O}}_{2}\text{ content }\times \text{ CaBF},$$

where:

$$\text{Blood }{\text{O}}_{2}\text{ content }=\;\left(\text{haemoglobin concentration }\times \text{ Sa}{\text{O}}_{2}\times \;1.36\right)\;+\;\left(\text{Pa}{\text{O}}_{2}\times \;0.003\right).$$

### Statistical methods

Blood flow data were analysed using 2-way repeated measures ANOVA with group (multiple SI, single SI and no SI) and time as factors. Post hoc comparisons between groups and time-points were performed using the Holm-Sidak test. Data are presented as mean ± SEM unless otherwise stated and p values of p≤0.05 were considered as statistically significant. Histological data were compared by Kruskal-Wallis ANOVA on ranks; data are presented as median scores and interquartile range.

## Results

Blood gases, ventilation parameters, heart rate and carotid blood pressure have been previously published [12]. Fetal blood gases show PaO_2_ was higher in the single SI lambs compared to the other groups (Table 1). There were no differences in blood gases at the end of asphyxia, before the onset of ventilation. SaO_2_ was significantly higher in the single SI group compared to the multiple SI group between 2.5–5 minutes after onset of ventilation. PaO_2_ was significantly higher in the single SI group between 5–10 minutes after onset of ventilation. There were no differences in PaCO_2_ between the groups.

**Table 1:** Blood gas status.

	SaO_2_			PaO_2_			PaCO_2_		
	multiple SI	single SI	no SI	multiple SI	single SI	no SI	multiple SI	single SI	no SI
Fetal	44 ± 23	79 ± 11	50 ± 17	26 ± 15^b^	33 ± 11^a^^c^	19 ± 6^b^	55 ± 19	64 ± 21	65 ± 24
BV	10 ± 7	11 ± 9	9 ± 9	6 ± 4	8 ± 10	7 ± 4	91 ± 26	124 ± 11	96 ± 34
1	17 ± 9	49 ± 37	23 ± 13	10 ± 5	22 ± 11	12 ± 5	97 ± 23	109 ± 35	97 ± 32
2.5	21 ± 14^b^	75 ± 35^a^	37 ± 33	13 ± 6	40 ± 30	19 ± 14	93 ± 19	77 ± 35	86 ± 44
5	37 ± 33^b^	83 ± 37^a^	50 ± 42	22 ± 22^b^	74 ± 48^a^^c^	36 ± 34^b^	83 ± 17	60 ± 39	83 ± 28
7.5	45 ± 40	81 ± 40	57 ± 42	33 ± 38^b^	82 ± 51^a^	66 ± 59	86 ± 26	59 ± 46	73 ± 34
10	50 ± 41	84 ± 36	67 ± 39	42 ± 50^b^	82 ± 50^a^	66 ± 52	74 ± 35	48 ± 42	67 ± 32
15	64 ± 36	94 ± 10	69 ± 40	49 ± 36	66 ± 31	69 ± 51	61 ± 25	45 ± 12	48 ± 32
20	68 ± 31	93 ± 11	71 ± 36	43 ± 28	71 ± 31	50 ± 29	57 ± 19	42 ± 12	51 ± 13
30	70 ± 29	86 ± 28	68 ± 38	36 ± 19	66 ± 14	39 ± 22	57 ± 16	41 ± 15	49 ± 18

BV, before onset of ventilation; SaO2, arterial oxygen saturation; PaO2, partial pressure of oxygen; PaCO2, partial pressure of carbon dioxide. Data are mean ± SD.

^‘a’^ p<0.05 vs multiple SI;

^‘b’^ p<0.05 vs single SI;

^‘c’^ p<0.05 vs no SI. Data adapted from previous publication [12].

**Fig 1:**
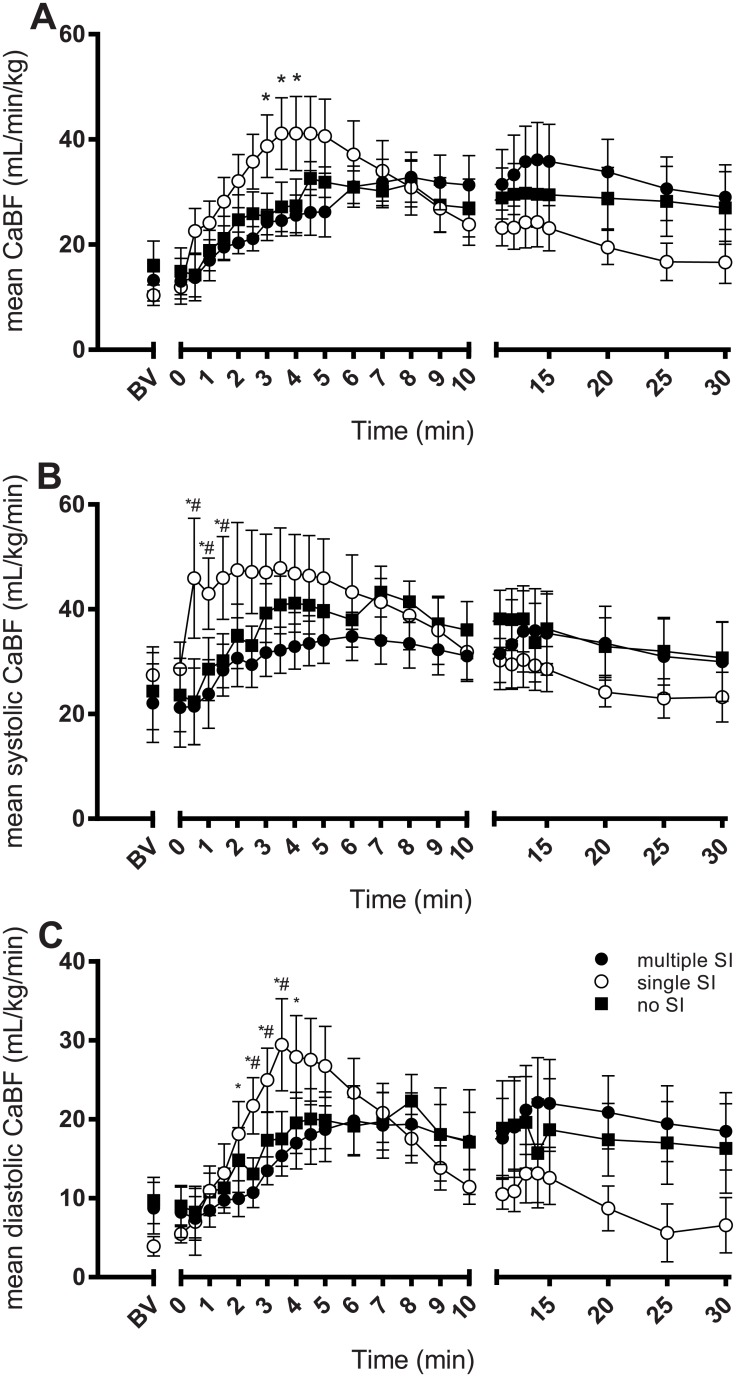
Arterial carotid blood flow. Mean carotid blood flow (CaBF; A), mean systolic CaBF (B), and mean diastolic CaBF (C) in lambs receiving 5 consecutive 3 s sustained inflations (multiple SI; ●), a single 30 s sustained inflation (single SI; ○) or conventional ventilation (no SI; ■) before the onset of ventilation (BV) and with the initiation of ventilation (designated as time 0). Data are mean ± SEM. * p<0.05 single SI vs multiple SI; # p<0.05 single SI vs no SI.

Mean CaBF in all groups was below 20 mL/kg/min at the end of the asphyxia period (Fig 1A). Mean CaBF in lambs given a single SI increased more rapidly, and to a greater extent (peak of 41.1 ± 7.0 mL/kg/min at 4 min after ventilation onset), compared to lambs given multiple SI (Fig 1A). CaBF in single SI lambs then returned to similar values as the other two groups by 5–6 min. Single SI lambs tended to have lower CaBF for the last 10 min of ventilation although this was not statistically significant.

Mean systolic CaBF in single SI lambs increased from 28.6 ± 5.1 to 45.9 ± 11.5 mL/kg/min at 30 sec after ventilation onset (Fig 1B). In contrast, mean systolic CaBF remained low in the multiple SI and no SI lambs for up to 1 min after ventilation onset. There were no differences between groups after 2 min of ventilation.

In single SI lambs, mean diastolic CaBF increased from 5.5 ± 1.1 to 27.9 ± 5.2 mL/kg/min by 4 min after ventilation onset, which was much greater compared to both other ventilation groups (Fig 1C). Following a peak at 3–4 mins after ventilation onset, mean diastolic CaBF gradually decreased in single SI lambs reaching similar levels as both other groups at 6–7 minutes. However, this decrease in mean diastolic CaBF continued for the remaining 20 min of ventilation, resulting in values that were below both other groups, although this was not statistically significant.

**Fig 2:**
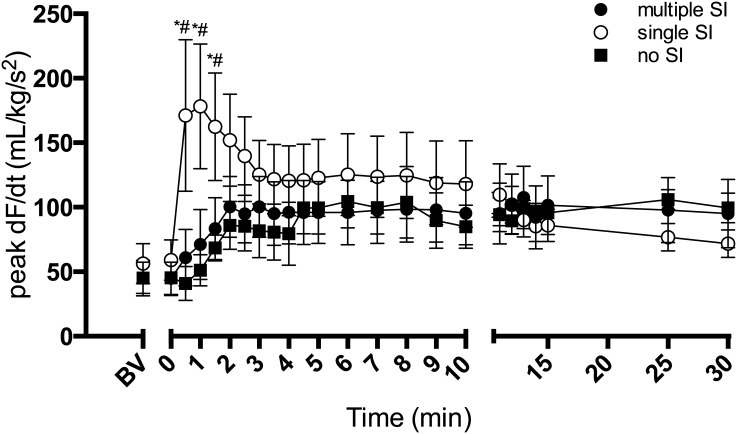
Peak dF/dT. Peak derivative of the carotid arterial blood flow pulse (peak dF/dT) in lambs receiving 5 consecutive 3 s sustained inflations (multiple SI; ●), a single 30 s sustained inflation (single SI; ○) or conventional ventilation (no SI; ■) before the onset of ventilation (BV) and with the initiation of ventilation (designated as time 0). Data are mean ± SEM. * p<0.05 single SI vs multiple SI; # p<0.05 single SI vs no SI.

The post-asphyxic increase in heart contractility (peak dF/dt of the CaBF wave form) was much greater in single SI lambs compared to both other groups (Fig 2). At 1 min from ventilation onset, heart contactility in single SI lambs (178.2 ± 48.4 mL/kg/s^2^) was more than double that of both multiple SI (71.1 ± 27.1 mL/kg/s^2^) and no SI lambs (51.1 ± 12.2 mL/kg/s^2^). Peak dF/dt then decreased in single SI lambs 1 min after ventilation onset. There were no differences between all groups after 2 min ventilation.

**Fig 3:**
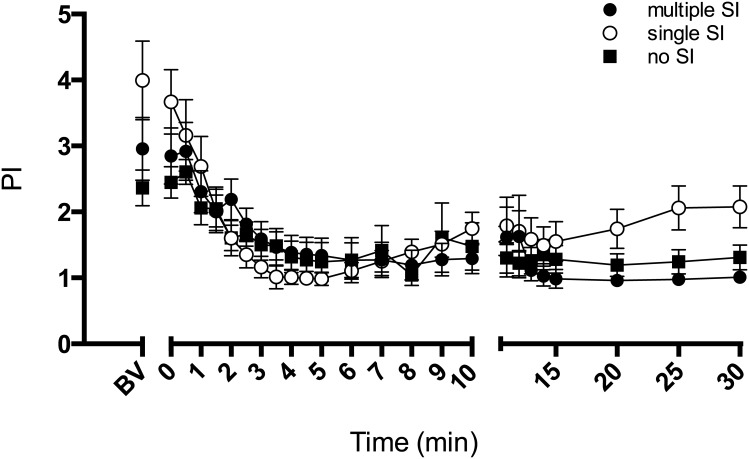
Pulsatility index. Pulsatility index of the carotid blood flow (PI) in lambs receiving 5 consecutive 3 s sustained inflations (multiple SI; ●), a single 30 s sustained inflation (single SI; ○) or conventional ventilation (no SI; ■) before the onset of ventilation (BV) and with the initiation of ventilation (designated as time 0). Data are mean ± SEM.

The CaBF PI decreased in all groups after ventilation onset (Fig 3). PI in lambs given multiple SI or no SI remained unchanged after 15 min of ventilation.

**Fig 4:**
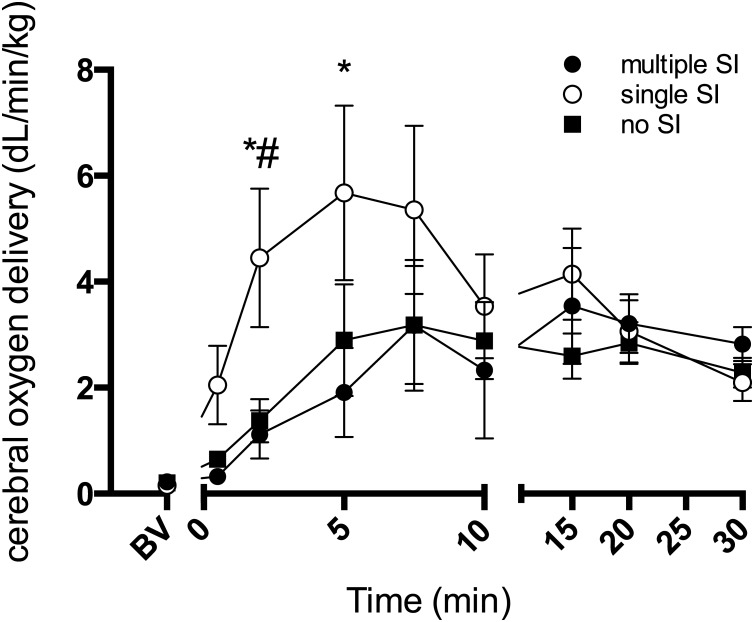
Cerebral oxygen delivery. Cerebral oxygen delivery in lambs receiving 5 consecutive 3 s sustained inflations (multiple SI; ●), a single 30 s sustained inflation (single SI; ○) or conventional ventilation (no SI; ■) before the onset of ventilation (BV) and with the initiation of ventilation (designated as time 0). Data are mean ± SEM. * p<0.05 single SI vs multiple SI; # p<0.05 single SI vs no SI.

Cerebral oxygen delivery, as estimated from carotid arterial oxygen content and CaBF, increased more rapidly and to a greater extent in single SI lambs immediately after ventilation onset compared to other groups (Fig 4). At 5 min of ventilation in single SI lambs, oxygen delivery was 5.7 ± 1.6 dL/kg/min, more than double that of both multiple SI (1.9 ± 0.8 dL/kg/min) and no SI lambs (2.8 ± 1.0 dL/kg/min). There were no differences in cerebral oxygen delivery between groups after 10 min of ventilation.

**Fig 5:**
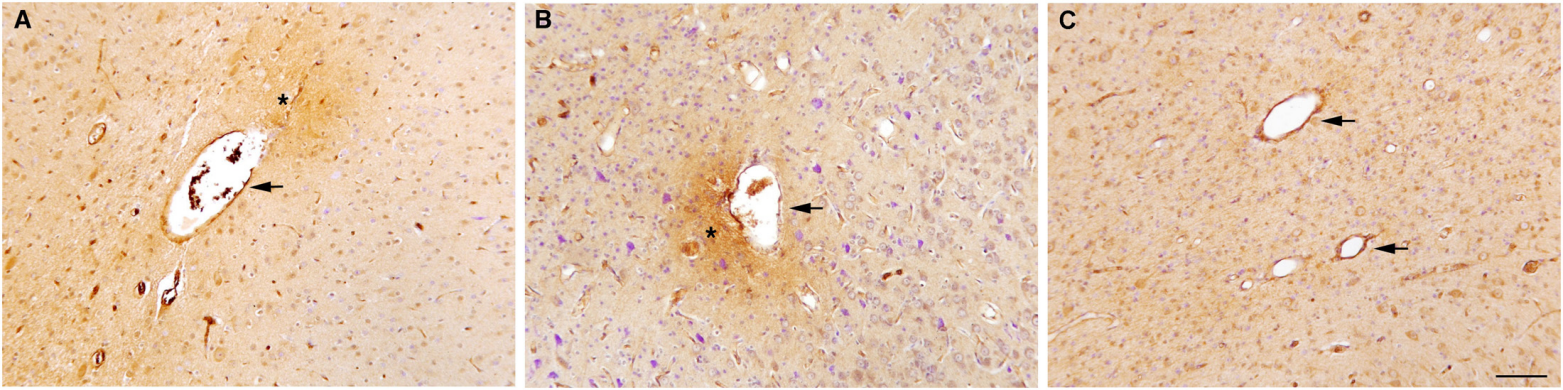
Representative images of blood vessel profiles. Representative images of blood vessel profiles (indicated by arrowheads) with serum extravasation (indicated by asterisk) in the subcortical white matter of a lamb that received a single 30 s sustained inflation (single SI; A) and 5 consecutive 3 s sustained inflations (multiple SI; B). Representative images of blood vessel profiles (indicated by arrowheads) with no serum extravasation in the subcortical white matter of a lamb that received no sustained inflation (no SI; C). Scale bar represents 90μm.

**Fig 6:**
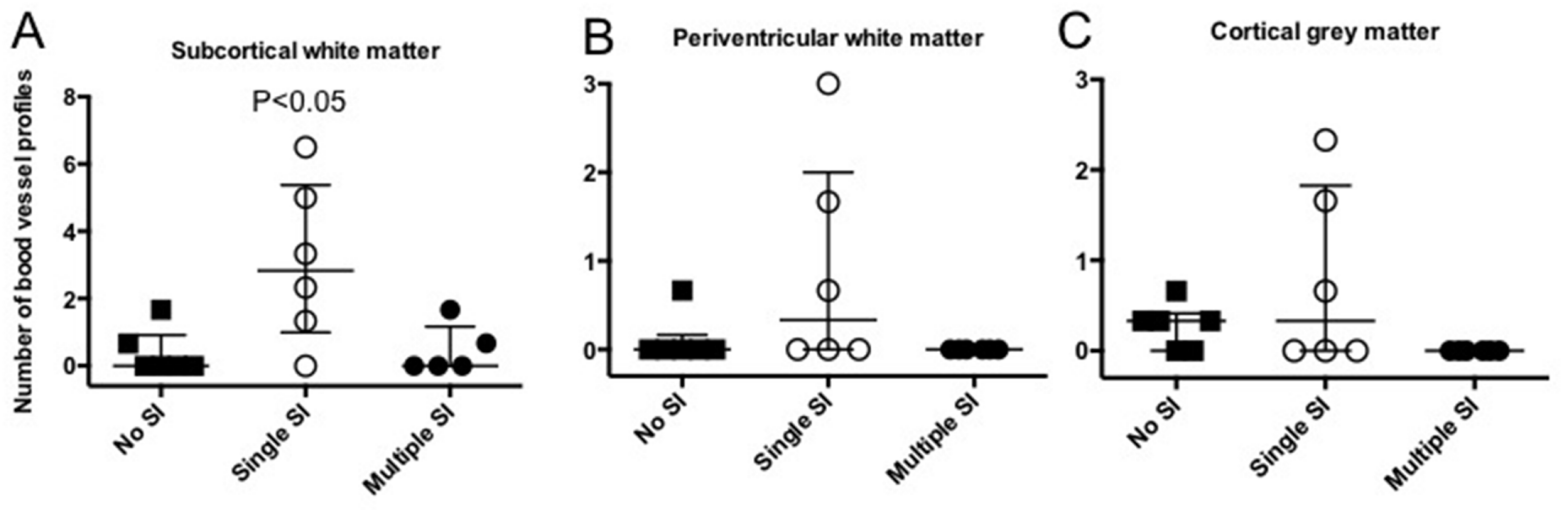
Blood vessel profiles. Number of blood vessel profiles with serum extravasation in the subcortical white matter (A), periventricular white matter (B) and cortical grey matter (C) in lambs that received conventional ventilation (no SI, n = 6; ■), a single 30 s sustained inflation (single SI, n = 6; ○) or 5 consecutive 3 s sustained inflations (multiple SI, n = 5; ●). Data presented as median (IQR). One animal in the multiple SI group was omitted from analysis due to poor tissue quality.

Serum extravasation, evident as small patches of sheep serum-immunoreactivity around capillaries, and disrupted blood vessels were observed in white and grey matter in 2/5 lambs in the multiple SI group, 6/6 in the single SI group and 4/6 in the no SI group; one animal in the multiple SI group was omitted from analysis due to poor tissue quality (Fig 5). The total number of disrupted blood vessels with serum extravasation was significantly increased (p< 0.05) in the subcortical white matter of single SI lambs compared to both other experimental groups (Fig 6A). However, in both the periventricular white matter (Fig 6B) and the cortical grey matter (Fig 6C), the number of blood vessels with serum extravasation was not different between groups although they tended to be elevated in single SI lambs. There was no evidence of overt brain injury (lesions, necrosis, hemorrhage) within the cerebral hemisphere in all lambs irrespective of experimental group.

## Discussion

We have previously shown that circulatory recovery is more rapid in severely asphyxic, bradycardic newborn lambs given an initial single 30 s SI compared to lambs given 5 consecutive 3 s SIs or conventional ventilation without a SI [12]. The results of this study extend these findings and demonstrate that CaBF, cerebral oxygen delivery and cardiac contractility also increase more rapidly and to a greater extent in asphyxiated near-term lambs given a single 30 s SI. However, plasma protein extravasation was observed in a greater number of blood vessel profiles in single SI lambs, indicating greater disruption to the blood brain barrier. This is possibly due to the size and speed of the cardiovascular response following a SI, which rapidly increased blood pressure, flows and heart rates from the severely depressed state that occurred during asphyxia.

The much higher CaBF during the first 5 min of ventilation in lambs given a single SI is likely due to increased cardiac contractility resulting from better lung aeration in these lambs [6, 20]. Lung aeration causes a marked increase in pulmonary blood flow and oxygenation [7], which increases pulmonary venous return and oxygen delivery to the heart [21]. This, in turn, improves the coronary circulation and cardiac function, increasing heart rate and contractility, as reflected by an increase in peak dF/dt during the first 2 min of ventilation. However, if this improvement in cardiac function is too rapid or unstable, it may result in large fluctuations in cerebral blood flow, thereby increasing the risk of cerebral vascular injury [22–24]. We did not measure venous pressure and, therefore, the effect of SI on central venous pressures or on the cerebral venous circulation are unknown.

Hypoxia and hypercapnia are potent cerebral vasodilators, which is an autoregulatory response designed to sustain blood flow and oxygen delivery to the brain [25]. As a result, the cerebral circulation was likely to have been maximally vasodilated in response to the asphyxia, thereby exposing the delicate cerebral microcirculation to high pressures and flow resulting from the rapid increases in cardiac function during the recovery phase. As asphyxia also impairs cerebral autoregulation, cerebral blood flow becomes pressure-passive and, as a result, the vulnerability of the brain to cardiovascular instability is markedly increased [23, 26]. Furthermore, this effect may have been enhanced by simultaneous peripheral vasoconstriction, which reduces the compliance of the peripheral circulation and reduces its ability to dampen the large pressure changes caused by increases in cardiac output. This contention is consistent with the finding that following an ischemic insult, fluctuations in cerebral blood flow increases the likelihood of cerebral vessel rupture and hemorrhage in preterm infants [27, 28]

To our knowledge, this is the first study to look at the effects of SIs on cerebral haemodynamics and subsequent blood brain barrier integrity in severely asphyxiated bradycardic animals. We observed an increased number of blood vessel profiles with plasma protein extravasation, a potential early marker of haemorrhage, in the single SI group. We were unable to confidently determine whether the extravasated blood vessels were veins or arteries, which would determine whether the leakage occurred directly due to arterial fluctuations or due to venous pooling. However, the finding of increased extravasation resultant from a single SI is consistent with findings in preterm infants. A recent study found increased incidence of mild IVH (23% vs 14%; p = 0.15) in preterm infants that received a SI [29] but there is no clarification as to whether those specific infants were asphyxic and bradycardic at birth. We did not see any evidence of venous congestions indicative of intracerebral or intraventricular haemorrhage or overt brain injury. As these lambs were only ventilated for 30 min, it is possible that this is insufficient time for gross morphological changes to manifest and be detected. However, as asphyxia by itself can impair cerebral autoregulation and disrupt the blood brain barrier [30, 31], it is possible that the severe asphyxial insult and the rapid cardiovascular responses induced by the single SI were cumulative, culminating in an aggravated injury response.

It is also possible that the increase in plasma protein extravasation detected in single SI lambs resulted from the subsequent ventilation these lambs received (data previously published [12]). Given the significant improvement in aeration and rapid decrease in lung compliance that resulted in lambs with a single SI, these lambs achieved much higher V_T_ (by 4–5 mL/kg) during the pressure controlled ventilation period than the other lambs. High V_T_ ventilation can significantly increase plasma protein extravasation in the white matter of preterm lambs, while ventilation after an initial SI using low V_T_ (7 mL/kg) prevented any extravasation [5]. However, no studies have yet investigated the association between SI administration and brain injury in asphyxiated newborn animals. Similarly, there has only been one study to show a potential increase in IVH [29] with SIs while two randomised trials and two retrospective studies comparing the use of SIs in the delivery room with standard treatment in preterm infants found no difference in IVH incidence [32–35]. Thus it is possible that the increase in extravasation resulted from the higher V_T_ after the recruitment strategy rather than the SI itself, but more studies are required to elucidate this finding. Thus, caution must be used when applying pressure limited ventilation after an SI, and volume targeting should be used where possible.

Following 5 min of ventilation after the single SI, cerebral oxygen delivery had increased 6-fold, which is consistent with what has been observed in very low birth weight human infants given an ‘effective’ SI. These infants also rapidly increased their heart rate and cerebral tissue oxygenation (measured using near infrared spectroscopy) [8]. The increase in cerebral oxygen delivery in single SI lambs is likely responsible for the subsequent decrease in CaBF observed at this time point. Although PI was not different between the groups, it began to increase in single SI lambs 5 min after ventilation, reflecting an increase in cerebral vascular resistance. This increase in cerebral vascular resistance, and associated reduction in CaBF, is a compensatory mechanism initiated by an intact ability to autoregulate cerebral blood flow, despite being delayed. Following an initial asphyxic insult, rapid reperfusion of blood flow and markedly increased oxygen delivery rates to the brain can further increase oxidative stress, blood brain barrier permeability and haemorrhaging [30, 31]. Therefore, although delayed, the decrease in CaBF after 5 min is potentially protective and may prevent further haemorrhage and brain injury caused by oxidative stress.

In this study we used of carotid arterial blood flow as a measure of cerebral blood flow. CaBF has been shown to be strongly correlated with cerebral blood flow in sheep [14, 15], has been assessed over a wide range of values [15] and has been used as a measure of global cerebral blood flow in asphyxic fetal sheep [36, 37]. While its validity in asphyxic newborn lambs has not been clarified, given its use in many animal and human studies as a surrogate for cerebral blood flow, as well as the inability of other techniques, such as microspheres, to measure cerebral blood flow continuously, it is the best approximation we have of cerebral blood flow. In view of our observa- tions, it would now be worthwhile examining whether there are region-specific changes in blood flow within the brain as a result of a SI, and exploring whether regional differences were associated with patterns of vascular leakage.

In conclusion, we have demonstrated that a single 30 s SI increases CaBF and cerebral oxygen delivery more rapidly and to a greater extent than 5 consecutive 3 s SIs or conventional ventilation. The changes to CaBF mirror the rapid increase in heart rate and arterial blood pressure previously shown [12]. However, it is possible that this response was so rapid that it resulted in a greater incidence of vascular disruption and plasma protein extravasation and higher rates of cerebral oxygen delivery, thereby increasing the risk of vascular injury and oxidative stress in these lambs. This injury may occur as a direct result of the SI, or to the subsequent higher V_T_ delivered during the subsequent period of pressure controlled ventilation. This study highlights the critical relationship between initial respiratory support in the delivery room and the underling cerebral pathology and the need for better monitoring during this critical period.

## Supporting Information

S1 Table Mean carotid blood flow (mL/kg/min) of individual lambs receiving 5 consecutive 3 s sustained inflations (multiple SI), a single 30 s sustained inflation (single SI) or conventional ventilation (no SI) before the onset of ventilation (BV) and with the initiation of ventilation. (PDF)

S2 Table Mean systolic carotid blood flow (mL/kg/min) of individual lambs receiving 5 consecutive 3 s sustained inflations (multiple SI), a single 30 s sustained inflation (single SI) or conventional ventilation (no SI) before the onset of ventilation (BV) and with the initiation of ventilation. (PDF)

S3 Table Mean diastolic carotid blood flow (mL/kg/min) of individual lambs receiving 5 consecutive 3 s sustained inflations (multiple SI), a single 30 s sustained inflation (single SI) or conventional ventilation (no SI) before the onset of ventilation (BV) and with the initiation of ventilation. (PDF)

S4 Table Peak dF/dt (mL/kg/s^2^) of individual lambs receiving 5 consecutive 3 s sustained inflations (multiple SI), a single 30 s sustained inflation (single SI) or conventional ventilation (no SI) before the onset of ventilation (BV) and with the initiation of ventilation. (PDF)

S5 Table Pulsality index of individual lambs receiving 5 consecutive 3 s sustained inflations (multiple SI), a single 30 s sustained inflation (single SI) or conventional ventilation (no SI) before the onset of ventilation (BV) and with the initiation of ventilation. (PDF)

S6 Table Cerebral oxygen delivery (dL/min/kg) of individual lambs receiving 5 consecutive 3 s sustained inflations (multiple SI), a single 30 s sustained inflation (single SI) or conventional ventilation (no SI) before the onset of ventilation (BV) and with the initiation of ventilation. (PDF)

## References

[1] Boyle DW, Szyld EG, Field D. Ventilation strategies in the depressed term infant. Seminars in fetal & neonatal medicine. 2008;13(6):392–400. Epub 2008/08/01. S1744-165X(08)00071-1 [pii] 10.1016/j.siny.2008.04.026 . 18667370

[2] Perlman JM, Wyllie J, Kattwinkel J, Atkins DL, Chameides L, Goldsmith JP, et al Part 11: Neonatal resuscitation: 2010 International Consensus on Cardiopulmonary Resuscitation and Emergency Cardiovascular Care Science With Treatment Recommendations. Circulation. 2010;122(16 Suppl 2):S516–38. Epub 2010/10/22. 122/16_suppl_2/S516 [pii] 10.1161/CIRCULATIONAHA.110.971127 . 20956259

[3] Polglase GR, Miller SL, Barton SK, Kluckow M, Gill AW, Hooper SB, et al Respiratory support for premature neonates in the delivery room: effects on cardiovascular function and the development of brain injury. Pediatr Res. 2014;75(6):682–8. 10.1038/pr.2014.40 . 24614803

[4] Polglase GR, Hooper SB, Gill AW, Allison BJ, McLean CJ, Nitsos I, et al Cardiovascular and pulmonary consequences of airway recruitment in preterm lambs. J Appl Physiol. 2009;106(4):1347–55. Epub 2009/02/14. 91445.2008 [pii] 10.1152/japplphysiol.91445.2008 . 19213936

[5] Polglase GR, Miller SL, Barton SK, Baburamani AA, Wong FY, Aridas JD, et al Initiation of resuscitation with high tidal volumes causes cerebral hemodynamic disturbance, brain inflammation and injury in preterm lambs. PloS one. 2012;7(6):e39535 Epub 2012/07/05. 10.1371/journal.pone.0039535 PONE-D-12-06915 [pii]. 22761816 PMC3382197

[6] te Pas AB, Siew M, Wallace MJ, Kitchen MJ, Fouras A, Lewis RA, et al Effect of sustained inflation length on establishing functional residual capacity at birth in ventilated premature rabbits. Pediatr Res. 2009;66(3):295–300. Epub 2009/06/23. 10.1203/PDR.0b013e3181b1bca4 . 19542905

[7] Sobotka KS, Hooper SB, Allison BJ, Te Pas AB, Davis PG, Morley CJ, et al An initial sustained inflation improves the respiratory and cardiovascular transition at birth in preterm lambs. Pediatr Res. 2011;70(1):56–60. Epub 2011/06/11. 10.1203/PDR.0b013e31821d06a1 00006450-201107000-00011 [pii]. . 21659961

[8] Fuchs H, Lindner W, Buschko A, Trischberger T, Schmid M, Hummler HD. Cerebral oxygenation in very low birth weight infants supported with sustained lung inflations after birth. Pediatr Res. 2011;70(2):176–80. Epub 2011/04/28. 10.1203/PDR.0b013e318220c1e0 . 21522035

[9] Richmond S, Wyllie J. European Resuscitation Council Guidelines for Resuscitation 2010 Section 7. Resuscitation of babies at birth. Resuscitation. 2010;81(10):1389–99. Epub 2010/10/20. S0300-9572(10)00444-2 [pii] 10.1016/j.resuscitation.2010.08.018 . 20956046

[10] Vyas H, Milner AD, Hopkin IE, Boon AW. Physiologic responses to prolonged and slow-rise inflation in the resuscitation of the asphyxiated newborn infant. The Journal of pediatrics. 1981;99(4):635–9. Epub 1981/10/01. . 7277110 10.1016/s0022-3476(81)80279-x

[11] Hoskyns EW, Milner AD, Hopkin IE. A simple method of face mask resuscitation at birth. Archives of disease in childhood. 1987;62(4):376–8. Epub 1987/04/01. 3592728 10.1136/adc.62.4.376 PMC1778339

[12] Klingenberg C, Sobotka KS, Ong T, Allison BJ, Schmolzer GM, Moss TJ, et al Effect of sustained inflation duration; resuscitation of near-term asphyxiated lambs. Archives of disease in childhood Fetal and neonatal edition. 2013;98(3):F222–7. Epub 2012/07/12. archdischild-2012-301787 [pii] 10.1136/archdischild-2012-301787 . 22782994

[13] Polglase GR, Wallace MJ, Grant DA, Hooper SB. Influence of fetal breathing movements on pulmonary hemodynamics in fetal sheep. Pediatr Res. 2004;56(6):932–8. . 15470203 10.1203/01.PDR.0000145254.66447.C0

[14] van Bel F, Roman C, Klautz RJ, Teitel DF, Rudolph AM. Relationship between brain blood flow and carotid arterial flow in the sheep fetus. Pediatric research. 1994;35(3):329–33. Epub 1994/03/01. . 8190521 10.1203/00006450-199403000-00011

[15] Covert RF, Schreiber MD, Torgerson LJ, Torgerson RW, Miletich DJ. Prediction of cerebral blood flow in fetal lambs by carotid artery ultrasonic flow transducer. Reproduction, fertility, and development. 1996;8(1):157–62. Epub 1996/01/01. . 8713735 10.1071/rd9960157

[16] Rees S, Hale N, De Matteo R, Cardamone L, Tolcos M, Loeliger M, et al Erythropoietin is neuroprotective in a preterm ovine model of endotoxin-induced brain injury. J Neuropathol Exp Neurol. 2010;69(3):306–19. Epub 2010/02/10. 10.1097/NEN.0b013e3181d27138 . 20142760

[17] Gosling RG, King DH. Arterial assessment by Doppler-shift ultrasound. Proc R Soc Med. 1974;67(6 Pt 1):447–9. Epub 1974/06/01. 4850636 10.1177/00359157740676P113 PMC1645777

[18] Mason DT, Braunwald E, Covell JW, Sonnenblick EH, Ross J Jr. Assessment of cardiac contractility. The relation between the rate of pressure rise and ventricular pressure during isovolumic systole. Circulation. 1971;44(1):47–58. Epub 1971/07/01. . 5561416 10.1161/01.cir.44.1.47

[19] Lister G, Walter TK, Versmold HT, Dallman PR, Rudolph AM. Oxygen delivery in lambs: cardiovascular and hematologic development. Am J Physiol. 1979;237(6):H668–H75. Epub 1979/12/01. . 517666 10.1152/ajpheart.1979.237.6.H668

[20] te Pas AB, Siew M, Wallace MJ, Kitchen MJ, Fouras A, Lewis RA, et al Establishing functional residual capacity at birth: the effect of sustained inflation and positive end-expiratory pressure in a preterm rabbit model. Pediatr Res. 2009;65(5):537–41. Epub 2009/02/05. . 19190537 10.1203/PDR.0b013e31819da21b

[21] Crossley KJ, Allison BJ, Polglase GR, Morley CJ, Davis PG, Hooper SB. Dynamic changes in the direction of blood flow through the ductus arteriosus at birth. The Journal of physiology. 2009;587(Pt 19):4695–704. Epub 2009/08/14. jphysiol.2009.174870 [pii] 10.1113/jphysiol.2009.174870 19675069 PMC2768022

[22] Hillman NH, Moss TJ, Kallapur SG, Bachurski C, Pillow JJ, Polglase GR, et al Brief, large tidal volume ventilation initiates lung injury and a systemic response in fetal sheep. American journal of respiratory and critical care medicine. 2007;176(6):575–81. Epub 2007/07/21. 200701-051OC [pii] 10.1164/rccm.200701-051OC 17641159 PMC1994225

[23] Lou HC, Lassen NA, Friis-Hansen B. Impaired autoregulation of cerebral blood flow in the distressed newborn infant. J Pediatr. 1979;94(1):118–21. Epub 1979/01/01. . 758388 10.1016/s0022-3476(79)80373-x

[24] Van Bel F, Walther FJ. Myocardial dysfunction and cerebral blood flow velocity following birth asphyxia. Acta Paediatr Scand. 1990;79(8–9):756–62. Epub 1990/08/01. . 2239269 10.1111/j.1651-2227.1990.tb11551.x

[25] Del Toro J, Louis PT, Goddard-Finegold J. Cerebrovascular regulation and neonatal brain injury. Pediatr Neurol. 1991;7(1):3–12. Epub 1991/01/01. 0887-8994(91)90098-6 [pii]. . 2029291 10.1016/0887-8994(91)90098-6

[26] Lou HC, Lassen NA, Tweed WA, Johnson G, Jones M, Palahniuk RJ. Pressure passive cerebral blood flow and breakdown of the blood-brain barrier in experimental fetal asphyxia. Acta Paediatr Scand. 1979;68(1):57–63. Epub 1979/01/01. . 31759 10.1111/j.1651-2227.1979.tb04430.x

[27] Perlman JM, McMenamin JB, Volpe JJ. Fluctuating cerebral blood-flow velocity in respiratory-distress syndrome. Relation to the development of intraventricular hemorrhage. The New England journal of medicine. 1983;309(4):204–9. Epub 1983/07/28. 10.1056/NEJM198307283090402 . 6866033

[28] Van Bel F, Van de Bor M, Stijnen T, Baan J, Ruys JH. Aetiological role of cerebral blood-flow alterations in development and extension of peri-intraventricular haemorrhage. Dev Med Child Neurol. 1987;29(5):601–14. Epub 1987/10/01. . 3311857 10.1111/j.1469-8749.1987.tb08502.x

[29] Grasso C, Sciacca P, Giacchi V, Carpinato C, Mattia C, Palano GM, et al Effects of Sustained Lung Inflation, a lung recruitment maneuver in primary acute respiratory distress syndrome, in respiratory and cerebral outcomes in preterm infants. Early human development. 2015;91(1):71–5. Epub 2015/01/01. 10.1016/j.earlhumdev.2014.12.002 . 25549915

[30] Kluckow M, Evans N. Low superior vena cava flow and intraventricular haemorrhage in preterm infants. Arch Dis Child Fetal Neonatal Ed. 2000;82(3):F188–94. Epub 2000/05/05. 10794784 10.1136/fn.82.3.F188 PMC1721081

[31] Fellman V, Raivio KO. Reperfusion injury as the mechanism of brain damage after perinatal asphyxia. Pediatr Res. 1997;41(5):599–606. Epub 1997/05/01. 10.1203/00006450-199705000-00001 . 9128279

[32] te Pas AB, Walther FJ. A randomized, controlled trial of delivery-room respiratory management in very preterm infants. Pediatrics. 2007;120(2):322–9. Epub 2007/08/03. 120/2/322 [pii] 10.1542/peds.2007-0114 . 17671058

[33] Lindner W, Hogel J, Pohlandt F. Sustained pressure-controlled inflation or intermittent mandatory ventilation in preterm infants in the delivery room? A randomized, controlled trial on initial respiratory support via nasopharyngeal tube. Acta paediatrica. 2005;94(3):303–9. Epub 2005/07/21. . 16028648 10.1111/j.1651-2227.2005.tb18431.x

[34] Lindner W, Vossbeck S, Hummler H, Pohlandt F. Delivery room management of extremely low birth weight infants: spontaneous breathing or intubation? Pediatrics. 1999;103(5 Pt 1):961–7. Epub 1999/05/01. . 10224173 10.1542/peds.103.5.961

[35] Lista G, Fontana P, Castoldi F, Cavigioli F, Dani C. Does sustained lung inflation at birth improve outcome of preterm infants at risk for respiratory distress syndrome? Neonatology. 2011;99(1):45–50. Epub 2010/07/10. 000298312 [pii] 10.1159/000298312 . 20616570

[36] Bennet L, Peebles DM, Edwards AD, Rios A, Hanson MA. The cerebral hemodynamic response to asphyxia and hypoxia in the near-term fetal sheep as measured by near infrared spectroscopy. Pediatr Res. 1998;44(6):951–7. Epub 1998/12/16. 10.1203/00006450-199812000-00022 . 9853934

[37] Fujii EY, Takahashi N, Kodama Y, Roman C, Ferriero DM, Parer JT. Hemodynamic changes during complete umbilical cord occlusion in fetal sheep related to hippocampal neuronal damage. American journal of obstetrics and gynecology. 2003;188(2):413–8. Epub 2003/02/20. . 12592249 10.1067/mob.2003.40

