# Correction: Single Sustained Inflation followed by Ventilation Leads to Rapid Cardiorespiratory Recovery but Causes Cerebral Vascular Leakage in Asphyxiated Near-Term Lambs

**DOI:** 10.1371/journal.pone.0156193

**Published:** 2016-05-18

**Authors:** Kristina S. Sobotka, Stuart B. Hooper, Kelly J. Crossley, Tracey Ong, Georg M. Schmölzer, Samantha K. Barton, Annie R. A. McDougall, Suzie L. Miller, Mary Tolcos, Claus Klingenberg, Graeme R. Polglase

The following information is missing from the Funding section: This work was supported by National Institute of Health R01HD072848-01A1.
