# Supplementary material for: Single Sustained Inflation followed by Ventilation Leads to Rapid Cardiorespiratory Recovery but Causes Cerebral Vascular Leakage in Asphyxiated Near-Term Lambs
Source: PLoS One. 2016 Jan 14;11(1):e0146574. doi: 10.1371/journal.pone.0146574 (PMC4713062; doi:10.1371/journal.pone.0146574)
Supplement: S1 Table — (PDF) [file pone.0146574.s001.pdf]

Table S1. Mean carotid blood flow (mL/kg/min) of individual animals in multiple SI, single SI and no SI groups from onset of ventilation.

	multiple SI								single SI								no SI							
time (min)	1	2	3	4	5	6	mean	SEM	1	2	3	4	5	6	mean	SEM	1	2	3	4	5	6	mean	SEM
BV	22.04	7.65	8.16	2.47	28.16	10.74	13.20	4.00	15.20	16.90	11.18	4.65	7.63	6.82	10.40	2.00	8.59	3.70	10.60	33.64	26.87	12.12	15.92	4.76
0.00	24.88	5.48	7.48	2.51	27.83	9.95	13.02	4.35	18.36	18.02	12.18	3.85	10.25	9.03	11.95	2.27	7.59	3.50	9.39	32.96	22.20	13.90	14.93	4.45
0.30	22.48	4.40	10.55	1.50	29.70	13.68	13.72	4.39	38.39	28.89	26.84	10.42	15.20	15.85	22.60	4.30	7.69	3.17	9.32	32.23	17.32	15.58	14.22	4.18
1.00	18.95	13.38	22.57	2.54	30.89	13.83	17.03	3.92	35.98	30.39	29.08	8.64	15.27	25.32	24.11	4.18	20.92	6.08	23.15	33.80	12.83	16.38	18.86	3.88
1.30	21.73	12.78	24.09	15.92	27.47	15.01	19.50	2.36	40.82	33.75	35.70	12.63	16.19	29.93	28.17	4.60	18.80	17.71	27.55	37.96	8.20	16.72	21.16	4.20
2.00	24.24	13.30	24.86	18.34	25.07	16.27	20.35	2.07	46.00	31.81	44.30	19.13	16.38	34.85	32.08	5.05	19.59	18.17	27.42	42.19		16.15	24.70	4.77
2.30	26.92	12.71	19.80	20.30	28.01	19.01	21.13	2.30	48.10	33.93	50.10	24.89	17.80	39.71	35.75	5.22	14.54	22.14	29.96	41.34	31.16	16.08	25.87	4.17
3.00	29.21	13.19	26.01	24.95	29.17	23.25	24.30	2.42	52.76	30.31	53.71	28.86	18.70	47.87	38.70	5.98	9.03	24.26	32.65	38.63	31.14	16.05	25.29	4.54
3.30	30.76	12.92	18.56	29.22	30.16	25.73	24.56	2.97	56.83	28.75	59.55	29.41	20.57	51.50	41.10	6.85	10.93	27.63	36.65	40.90	30.14	16.74	27.16	4.69
4.00	34.32	12.75	14.67	32.81	29.77	29.21	25.59	3.84	59.15	26.84	58.20	28.68	21.48	52.55	41.15	7.05	7.30	30.19	38.30	40.54	29.66	18.14	27.36	5.14
4.30	35.22	12.53	12.86	34.89	29.10	32.11	26.12	4.34	60.25	25.56	57.78	28.54	22.80	51.88	41.14	7.06	37.86	31.84	39.14	39.55	27.38	19.78	32.59	3.22
5.00	34.72	12.35	10.68	36.07	29.63	33.91	26.23	4.74	60.02	24.44	55.56	27.28	23.07	53.25	40.60	7.09	33.15	33.52	39.26	38.51	25.55	21.26	31.88	2.92
6.00	37.37	11.98	34.77	35.47	30.30	36.39	31.05	3.94	55.45	21.97	48.31	24.50	22.53	49.92	37.11	6.39	33.37	34.09	38.23	37.09	21.47	21.21	30.91	3.12
7.00	37.81	11.13	41.04	33.57	30.72	36.64	31.82	4.38	49.58	19.73	43.81	22.33	21.57	46.99	34.00	5.78	34.27	33.00	37.47	35.82	21.27	19.37	30.20	3.19
8.00	37.70	11.27	44.95	29.84	34.84	38.35	32.82	4.76	45.39	16.94	41.07	19.97	20.67	41.15	30.87	5.28	30.14	29.71	36.19	43.96		17.56	31.51	4.34
9.00	36.28	10.37	47.83	26.15	32.22	38.21	31.84	5.19	42.95	14.93	36.28	19.00	20.14	28.02	26.89	4.47	24.66	27.06	34.36	48.75	13.18	17.12	27.52	5.23
10.00	33.95	10.35	51.16	22.96	33.13	36.43	31.33	5.60	37.56	15.98	34.64	15.22	18.56	21.14	23.85	3.98	19.28	23.34	32.50	51.95	18.31	16.57	26.99	5.51
11.00	34.96	4.99	50.60	21.44	42.04	34.77	31.47	6.59	33.13	26.39	31.59	12.98	16.86	18.06	23.17	3.42	20.92	20.90	31.13	55.29	28.12	16.97	28.89	5.69
12.00	42.27	4.23	49.84	18.82	50.71	33.66	33.25	7.55	29.48	38.64	26.45	12.79	15.61	16.37	23.22	4.09	20.91	19.03	28.67	54.38	37.55	16.97	29.59	5.85
13.00	48.11	15.79	48.92	16.77	52.49	32.71	35.80	6.77	27.79	43.72	29.94	12.13	15.34	16.46	24.23	4.87	21.35	16.75	25.76	52.73	42.58	19.58	29.79	5.92
14.00	52.77	15.17	47.70	17.00	53.00	31.22	36.14	7.13	27.43	43.45	29.47	13.05	15.05	17.15	24.27	4.71	20.22	15.68	22.77	50.74	47.44	20.54	29.56	6.26
15.00	53.60	15.29	46.45	16.94	52.22	30.33	35.80	7.09	26.42	40.62	27.80	12.80	14.18	17.06	23.15	4.33	18.94	16.59	20.94	49.10	50.41	20.84	29.47	6.45
20.00	53.26	15.90	41.83	21.42	46.17	24.23	33.80	6.22	21.10	33.08	20.35	11.18	11.35	19.93	19.50	3.28	17.15	25.22	16.64	49.50	44.22	20.15	28.81	5.88
25.00	51.51	16.88	36.98	19.60	41.97	16.88	30.64	6.07	19.15	32.51	13.44	11.32	7.33	16.65	16.73	3.57	15.75	22.27	16.18	51.68	46.19	17.33	28.23	6.65
30.00	50.11	16.79	33.01	17.32	42.30	14.58	29.02	6.14	16.96	34.92	15.22	11.11	5.89	15.62	16.62	4.01	15.22	20.25	16.15	52.25	44.65	13.43	26.99	6.92

BV, before ventilation; SEM, standard error of the mean; SI, sustained inflation
